# The Association between Acculturation and Dietary Patterns of South Asian Immigrants

**DOI:** 10.1371/journal.pone.0088495

**Published:** 2014-02-18

**Authors:** Iris A. Lesser, Danijela Gasevic, Scott A. Lear

**Affiliations:** 1 Department of Biomedical Physiology and Kinesiology, Simon Fraser University, Vancouver, British Columbia, Canada; 2 Faculty of Health Sciences, Simon Fraser University, Burnaby, British Columbia, Canada; 3 Division of Cardiology, Providence Health Care, Vancouver, British Columbia, Canada; CUNY, United States of America

## Abstract

Dietary acculturation, specifically the adoption of western dietary habits, may result in adverse health effects such as obesity and type 2 diabetes. Therefore, it is necessary to explore the role of acculturation in dietary patterns as well as awareness and knowledge of healthy nutrition among South Asian immigrants. This is an especially important population to target as South Asians have higher prevalence rates of type 2 diabetes and cardiovascular disease, which may be magnified with immigration. The current investigation is a sub-study of the Multi-Cultural Community Health Assessment Trial (M-CHAT). There were 207 participants of South Asian origin included in the initial study, 129 were born outside of Canada and had immigrated after the age of 18. The length of residence in Canada was used as a marker for acculturation. A questionnaire addressing perceived changes in dietary patterns, food preparation, and nutrition knowledge and awareness since immigration was used to assess dietary practices. The association between length of residence and variables related to perceived changes in dietary patterns was explored with Spearman correlation and significant associations were subsequently analyzed with ordinal logistic regression analysis adjusted for age, sex, education and body mass index. South Asian immigrants in Canada reported a variety of positive dietary practices, including an increased consumption of fruits and vegetables and an improvement in food preparation (including an increase in grilling and a decrease in deep frying when cooking). However, there was a reported increase in the consumption of convenience foods, sugar-sweetened beverages, red meat and in dining out. South Asian immigrants in Canada reported a variety of positive dietary practices including an improvement in food preparation. Future health promotion strategies should encourage cultural sensitivity in efforts to reduce the consumption of sugar-sweetened beverage, convenience foods and to encourage eating at home rather than dining out.

## Introduction

South Asians (populations originating from Bangladesh, India, Nepal, Pakistan and Sri Lanka) are one of the fastest growing immigrant populations in Canada [1] and the US [2]. Between 2001 and 2006, the South Asian population increased by more than 35% to 1.3 million, while the general Canadian population increased by only 5.4%. By 2031 it is expected that the Canadian South Asian population will nearly triple to 3.6 million [1], in addition, in the US the Asian population increased four times faster from 2000 to 2010 than the total US population, growing by 43% from 10.2 to 14.7 million [3]. The overwhelming majority of this growth will be from immigration as the growth of immigrant populations outpaces that of the native born populations [1].

It has been suggested that there exists a “healthy immigrant effect” where immigrants at the time of migration are in substantially better health than the native-born people in their new country. For instance, a lower prevalence of cardiovascular disease (CVD) risk factors has been reported among immigrants in their new country when compared to individuals in their country of origin [4]–[6]. This is due to immigration policies which require good health to qualify for immigration; however, it has been shown that this “healthy immigrant effect” deteriorates with length of residence in a new country [7]–[9] with immigrant health risk eventually surpassing that of native born populations [10].

This deterioration in the “healthy immigrant effect” may be due to the changes experienced in cultural, socio-economic, psychosocial, lifestyle and social support network changes with immigration which can have a negative impact on health [11]. Among those, dietary acculturation is a well established immigrant effect which denotes the process by which minority groups adopt the nutritional practice and diet of their host country. For example, it has recently been shown among Chinese immigrants that longer length of residence was associated with an increase in portion sizes and greater consumption of convenience food [12], and South Asian immigrants in the US showed an increase in the consumption of soft drinks, fruit juice and chips with longer length of residence [13]. Consequently, dietary acculturation, specifically the adoption of Western dietary habits, may result in adverse health effects such as obesity [14] and type 2 diabetes [15]. Therefore, it is necessary to explore the role of acculturation in dietary patterns as well as awareness and knowledge of healthy nutrition among South Asian immigants. This is an especially important population to target health promotion efforts as South Asians have higher prevalence rates of type 2 diabetes and CVD which may be magnified with immigration. Understanding the role of dietary acculturation will allow for the development of culurally sensitive nutrition programming to new immigrants in order to prevent cardiovascular disease in the South Asian population.

## Methods

All participants provided informed consent and this study was approved by the Simon Fraser University Research Ethic Board. The current investigation is a sub-study of the Multi-Cultural Community Health Assessment Trial (M-CHAT). Details on the study and participant recruitment have been published elsewhere [16]. Briefly, the M-CHAT study is a longitudinal community based study cohort of apparently healthy 30–65 year old men and women. Participants were recruited by multiple methods such as local media advertisements, community events and notices placed at community centres and cultural organizations. Individuals were considered to be of South Asian background if all ancestors originated from Bangladesh, India, Nepal, Pakistan and/or Sri Lanka based on self-report. There were 207 participants of South Asian origin included in the initial study, 129 were born outside of Canada and had immigrated after the age of 18 and 121 of the 129 participants completed the nutrition questionnaires. Fluency in English was not a requirement for participation.

All participants completed an interview for socio-demographics covering items such as age, sex, ethnicity, marital status, maximal educational level attained and annual household income. The length of residence in Canada was used as a marker for acculturation. Length of residence was obtained by subtracting age derived from birth date from self-reported age at the time of arrival in Canada. Weight in kilograms and height in metres were assessed with participants in light clothing (or hospital gowns), footwear removed and pockets emptied and body mass index was calculated. Participants completed a comprehensive questionnaire on the knowledge, beliefs and awareness of health, as well as health behaviours, that was developed based on the findings of earlier qualitative interviews held with our target communities, in combination with previously reported questionnaires. The present cross-sectional investigation focuses on the part of the questionnaire addressing perceived changes in dietary patterns, food preparation, and nutrition knowledge and awareness since immigration. Some examples of questions asked are, “How has your method of food preparation changed since coming to Canada?” and “Has your interest in information about the food you eat, such as ingredients, nutrition information and taking note of food labels, changed since coming to Canada?” All questions were based on a 5-point Likert-scale, with frequency results shown in three categories as either, decreased change/less often/harder (combined responses of 1 and 2), no change (response 3) or ‘increased change/more often/easier (combined responses of 4 and 5). Length of residence in Canada (time since immigration) was categorized into quartiles (cut off points: ≤13.8, 13.9–21.1, 21.2–32.1, and ≥32.2 years).

Data are presented as means ± standard deviation if continuous, and as counts and percentages if categorical. Differences in socio-demographic characteristics across quartiles of length of residence were explored by ANOVA and Chi-square test for continuous and categorical variables, respectively. Post hoc analyses were completed to explore inter-group differences. All food question items are presented as counts and percentages. The association between length of residence and variables related to perceived changes in dietary patterns was explored with Spearman correlation. Variables significantly associated with length of residence were subsequently analyzed with ordinal logistic regression analysis adjusted for age, sex, education and body mass index. The dependent variable was the food variable of interest using the original five categories of possible answers (‘much less’, ‘less’, ‘no change’, ‘more’ and ‘much more’) and the independent variable was the length of residence categorized in quartiles. A p-value of less than 0.05 was considered as statistically significant. Statistical analyses were performed using Statistical Package for Social Sciences (IBM, SPSS, version 19).

## Results

Table 1 summarizes participants' socio-demographic and anthropometric characteristics. Individuals in the 3^rd^ and 4^th^ quartile of immigration (who, by definition, had been in Canada longer) were significantly older than those in the 1^st^ quartile of immigration (p = <0.001). Those in the 2^nd^ quartile had a significantly higher BMI than those in the 3^rd^ quartile of immigration (p = 0.023) while there was no other significant difference amongst the quartiles of immigration for BMI (p<0.05). There were no significant differences in sex or education across quartiles of length of residence.

**Table 1:** Socio-demographic characteristics of South Asian participants across length of residence.

Length of Residence (years)	Quartile 1 ≤13.8 (n = 32)	Quartile 2 13.9–21.1 (n = 32)	Quartile 3 21.2–32.1 (n = 32)	Quartile 4 ≥32.2 (n = 25)	P value
Age (years)^a^	46.0±7.9	43.8±6.8	49.1±6.2	53.4±7.1	<0.001
Females	12 (37.5%)	13 (40.6%)	20 (60.6%)	17 (53.1%)	0.212
Maximal Educational Level Attained^b^					0.302
High school graduation or less	5 (15.5%)	52 (46.9%)	13 (39.4%)	13 (40.6%)	
Some post secondary education	11 (34.4%)	4 (12.5%)	4 (12.1%)	4 (12.5%)	
Post secondary or post graduate education	16 (50.1%)	13 (40.6%)	16 (48.5%)	15 (46.9%)	
Body mass index (BMI, kg/m^2^)^a^	28.9±5.4	29.8±4.7	26.5±4.2	27.1±3.5	0.012

a Mean ± SD.

b n (%).

**Figure 1:**
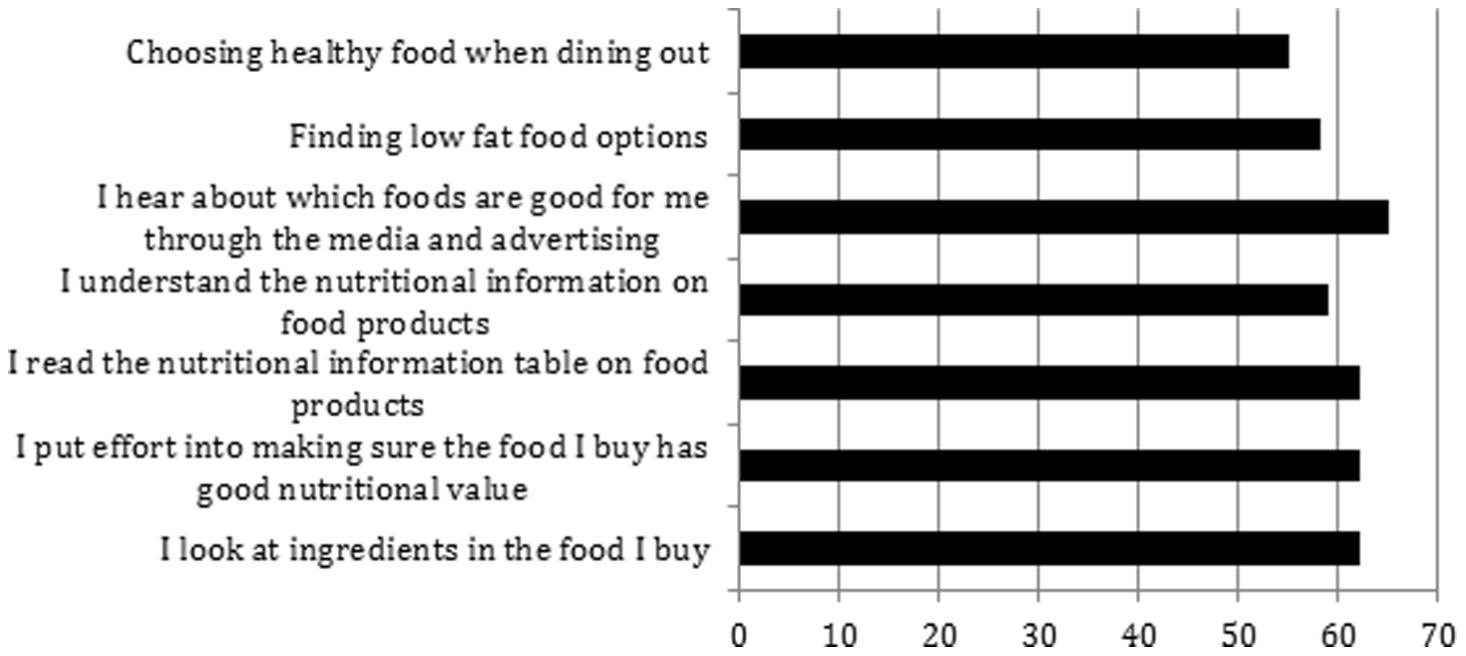
Percentage of participants reporting an improvement in food variables of interest indicative of positive changes since immigration (only variables with greater than 50% reported).

Figure 1 presents participant responses regarding the change in their interest about nutritional information such as ingredients and food labels since time of immigration. Seventy percent of South Asian immigrants reported receiving more information about healthy foods through media and advertisements with immigration to Canada. Nearly 65% of South Asian immigrants made an effort to purchase nutritional food after immigrating to Canada and 60% reported greater reading and understanding of nutritional information tables and ingredients on food products post immigration. Similarly, over 60% of South Asian immigrants found it easier to purchase low fat foods and almost 50% of immigrants found it easier to purchase fruits and vegetables in Canada compared to their homeland.

**Figure 2:**
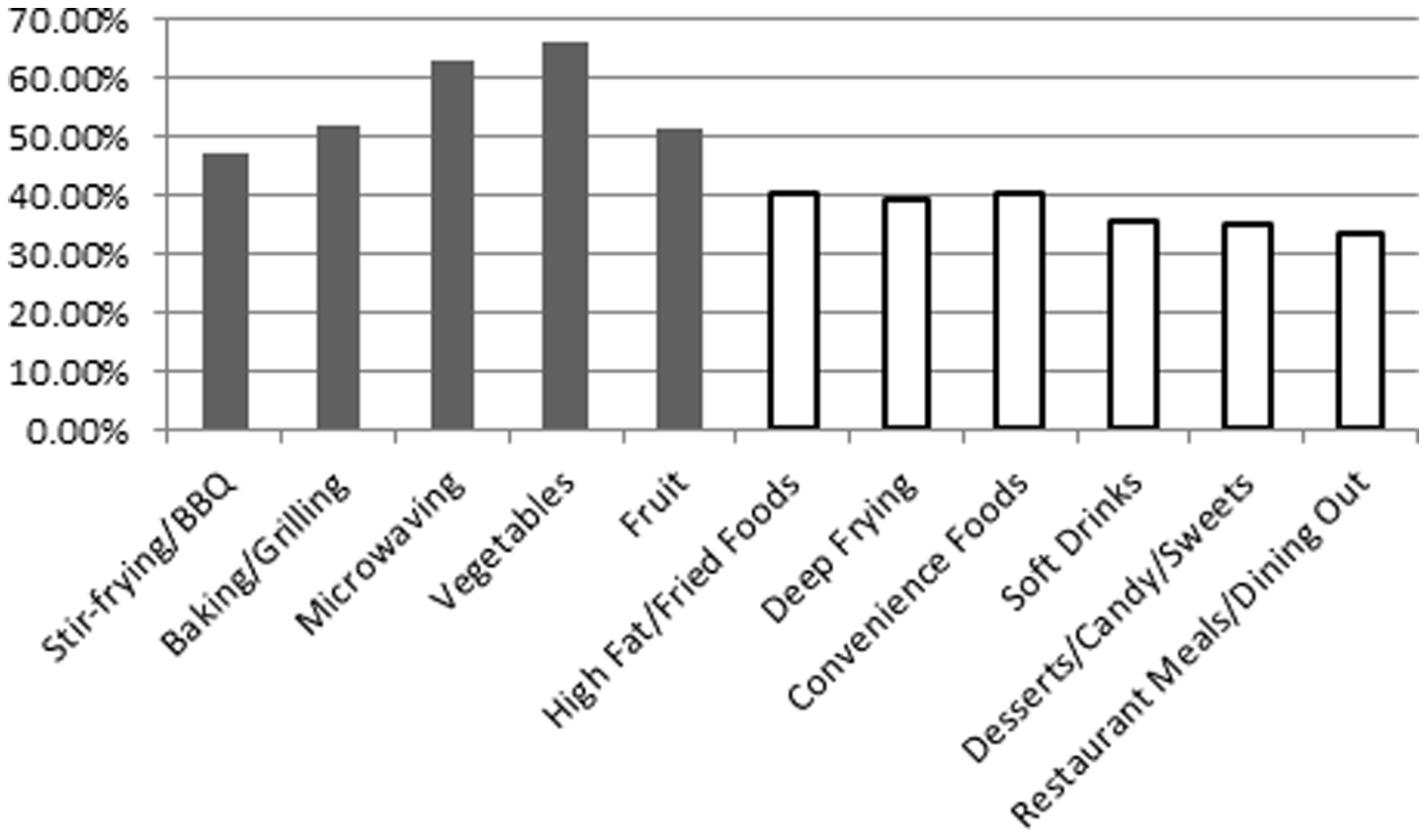
Percentage of participants reporting changes in food items and preparation since immigration (only variables with greater than 30% reported). Open bars represent negative dietary changes.

When dining out, nearly 60% of South Asian immigrants reported choosing healthier food options. Furthermore, changes were observed with regards to food consumption and preparation. Specifically, more than 60% of South Asians increased their consumption of fruits and vegetables since immigration to Canada, while consumption of high fat/fried food decreased among more than 40% of South Asian immigrants (Figure 2). Moreover, more than 50% of South Asians reported an increase in stir frying/BBQ and baking/grilling food while 40% of them decreased the amount of deep frying after immigrating to Canada (Figure 2). Compared to their homeland, South Asians reported that they consume more convenience foods (40.5%), soft drinks (35.5%), desserts/candy (34.9%), and they dine out more often (33.3%) (Figure 2).

Using Spearman's correlation, length of residence was positively associated with increased stir fry/BBQ (r = 0.201, p = 0.027), baking/grilling (r = 0.302, p = 0.001), microwaving (r = 0.181, p = 0.047) and consumption of red meat (r = 0.201, p = 0.027) (Table 2).

**Table 2:** Significant associations between length of residence and food variables of interest.

	Spearman Correlation	P value
Stir Fry or BBQ	0.201	0.027
Baking/Grilling	0.302	0.001
Microwaving	0.181	0.047
Red Meat Consumption	0.201	0.027

The results of ordinal regression showed that, compared to most recent immigrants, those in the second quartile of length of residence had an increased odds of stir frying or BBQ (OR (95%CI): 3.71 (1.19, 11.52)) and baking or grilling (OR (95% CI): 6.36 (1.95, 20.78)). Additionally, compared to most recent immigrants those in the third quartile of length of residence had an increased odds of microwaving (OR (95% CI): 2.75 (1.01, 7.43)) (Table 3). Length of residence was not significantly associated with any of the other outcomes assessed.

**Table 3:** Ordinal regression results of the association between acculturation and dietary items after adjustment for age, sex, education and body mass index.

Stir Fry or BBQ		
Length of Residence (years):	Odds Ratio (95% C.I)	P value
≤13.8	Reference	
13.9–21.1	3.71 (1.19, 11.52)	0.024
21.2–32.1	2.26 (0.85, 5.99)	0.101
≥32.2	1.01 (0.40, 2.57)	0.988

Bake or Grill		
Length of Residence (years):	Odds Ratio (95% C.I)	P value
≤13.8	Reference	
13.9–21.1	6.36 (1.95, 20.78)	0.002
21.2–32.1	2.49 (0.92, 6.73)	0.072
≥32.2	1.24 (0.48, 3.22)	0.654

Microwave		
Length of Residence (years):	Odds Ratio (95% C.I)	P value
≤13.8	Reference	
13.9–21.1	2.03 (0.65, 6.28)	0.221
21.2–32.1	2.75 (1.01, 7.43)	0.047
≥32.2	1.25 (0.49, 3.17)	0.643

Red Meat Consumption		
Length of Residence (years):	Odds Ratio (95% C.I)	P value
≤13.8	Reference	
13.9–21.1	2.77 (0.87, 8.90)	0.086
21.2–32.1	1.62 (0.59, 4.40)	0.347
≥32.2	0.54 (0.20, 1.42)	0.210

All analyses were adjusted for age, sex, education and BMI.

## Discussion

The results of our study indicate that, after immigration to Canada, the majority of South Asians reported an improvement in dietary practices. It is noteworthy that although not significant, BMI was lower with longer time since immigration, which may be related to this improvement in dietary practices. This includes an increase in consumption of fruits and vegetables and a reduction in intake of high fat/fried food. Moreover, reported changes to food preparation were primarily positive with an increase in healthier food preparation practices such as grilling and stir-frying, and a reduction in unhealthy food preparation practices such as deep-frying. Despite these positive changes, more than one third of respondents reported an increase in the consumption of convenience foods, soft drinks and desserts/candy as well as an increase in dining out. South Asian immigrants also reported a higher nutrition awareness facilitated by the availability of nutrition information through labelling or the promotion of good nutrition through the media and advertising. However, increased nutritional awareness and healthier food choices were not significantly associated with length of residence. In contrast, odds of stir frying/BBQ and baking/grilling among immigrants residing in Canada between 14 and 21 years were, respectively, about 4 and 6 times higher compared to their counterparts residing in Canada for less than 14 years. In addition, odds of microwaving food were about 3 times higher among South Asians who had immigrated 21 to 32 years ago compared to most recent immigrants. This suggests that length of residence since immigration may be important in food habits but it is unclear whether a specific duration of time since immigration is of importance.

Our study is consistent with previously published evidence on change in dietary practices among South Asians upon immigrating to Western countries. Namely, positive increases in dietary practices have been previously reported amongst South Asian immigrants in Norway including a 50% increase in fruit and vegetable consumption [17]. This study also reported that South Asian immigrants with a higher education consumed lower amounts of fat while those immigrants who had poor language skills consumed higher amounts of fat suggesting that health awareness and understanding may be important in immigrant diet. Additionally, immigrants from India to Eastern Canada reported that they were very or somewhat likely to engage in healthy lifestyle practices, were interested in nutrition information, and the majority indicated that they had changed their food preparation methods since immigration [18]. A reduction in the consumption of ghee and butter with length of residence in South Asian immigrants in the US was proposed by the authors to be due to an increase in the knowledge of the negative effects of saturated fats [13]. Specifically, the majority of respondents in this study reported that they were very or somewhat likely to read nutrition labels, avoid deep-frying, bake and BBQ more frequently and to consume more fruits and vegetables.

Other immigrant populations have also reported positive changes in dietary patterns. Arab immigrants in Canada and the US perceived their diet to be healthier after immigration due to increased nutritional awareness, differences in food preparation methods and the maintenance of cultural dietary practices [19], and greater access to healthy food choices [20]. Additionally, our group has previously reported that Chinese immigrants in Canada reported some favourable changes in dietary intake and greater awareness and more knowledge about foods after immigration [12]. Korean immigrants in the US were reported to have an increase in both positive and negative dietary habits with acculturation [21]. Pakistani immigrant women in Norway reported their nutritional habits to change with length of residence due to increased availability of nutritional knowledge [22]. The reported improvements in nutritional awareness in our population of South Asian immigrants as well as those observed in the above literature may be due to different requirements in Western countries compared to their country of origin concerning the labelling of food products as well as public health initiatives which promote healthy eating in Western countries.

While many positive changes to dietary practices have been observed among South Asian immigrants, there was an increase in convenience food, soft drinks, desserts/candy and dining out. The consumption of convenience foods and sugar sweetened beverages is commonly reported amongst migrants with the adoption of local foods due to their easy access and low cost [14], [18], [23] and has been denoted to primarily change with acculturation, as intake of these items is driven by taste preference and not tied to cultural identity [24]. Furthermore, we have also observed a significant association between red meat consumption and length of residence, however, this was not significant after adjustment for age, sex, education and body mass index. This is in congruence with the study from Norway where South Asians who have resided in Norway for more than ten years, have reported an increase in red meat consumption [17].

Cardiovascular disease and type 2 diabetes have strong associations with lifestyle, specifically dietary intake. In addition these diseases are more prevalent in the South Asian immigrant population. Our findings suggest that this increasing disease risk may be partially explained by the adoption of Western dietary practices. There was an increase in the consumption of red meat with time since immigration and vegetarianism is thought to be protective against myocardial infarction in the South Asian population [25] suggesting that the increase in red meat consumption may increase CVD risk [26]. A substantial percentage of South Asian immigrants that reported an increase in the consumption of convenience foods, sugar sweetened beverages and desserts/candy in our study and these dietary practices have been linked to obesity, type 2 diabetes and CVD [27]. An increase in dining out and convenience food consumption was reported in our population and these dietary practices were associated with obesity in a study on Hong Kong adults [28]. Although we did not see an increase in BMI in our population these negative dietary practices may explain the higher prevalence of type 2 diabetes and CVD among immigrants in their new country when compared to individuals in their native country [4]–[6].

Our study offers insight into the effects of acculturation on South Asian immigrants, however the following limitations should be acknowledged. Firstly, length of residence was used as a proxy indicator for acculturation while other measures of acculturation such as cultural attachment were not examined. In addition, data were based on self-report and we did not measure dietary intake. Self-report data, specifically data regarding diet, can be affected by participant bias and is an important limitation. In addition, our sample size was based on the number of participants of a larger study who were South Asian and born outside of Canada resulting in a smaller sample size. It is therefore necessary to replicate these results in a larger population sample. However, given the paucity of data in this area of research we feel that this study is highly valuable. Lastly, given the cross sectional nature of the study, we were unable to establish the causal relationship between acculturation and dietary practices. Consequently, longitudinal studies are needed to examine where there is a causal relationship between acculturation and changes in dietary practices among South Asian immigrants.

In conclusion, South Asian immigrants in Canada reported a variety of positive dietary practices, including an increased consumption of fruits and vegetables and an improvement in food preparation (including an increase in grilling and a decrease in deep frying when cooking). However, there was a reported increase in the consumption of convenience foods, sugar-sweetened beverages, red meat and in dining out. Future health promotion strategies should encourage cultural sensitivity in efforts to reduce the consumption of sugar-sweetened beverage, convenience foods and to encourage eating at home rather than dining out. Additional efforts should continue to focus on engaging this community in maintaining positive dietary behaviours.
